# Sensitivity and resistance to glucocorticoids

**DOI:** 10.1186/1546-0096-12-S1-I13

**Published:** 2014-09-17

**Authors:** George Chrousos

**Affiliations:** 1 Pediatrics, University of Athens, Athens, Greece

## 

In humans, glucocorticoids regulate a broad spectrum of physiologic functions essential for life and play an important role in the maintenance of basal and stress-related homeostasis. Approximately 20% of the genes expressed in human leukocytes are regulated positively or negatively by glucocorticoids. These steroids are involved in almost every cellular, molecular and physiologic network of the organism and play a pivotal role in critical biologic processes, such as growth, reproduction, intermediary metabolism, immune and inflammatory reactions, as well as central nervous system and cardiovascular functions. Physiologic amounts of glucocorticoids are also essential for normal renal tubular function and thus for water and electrolyte homeostasis. Furthermore, glucocorticoids represent one of the most widely used therapeutic compounds often employed in the treatment of inflammatory, autoimmune and lymphoproliferative disorders. Both excess and deficiency of glucocorticoids are respectively associated with disease, i.e. Cushing syndrome or Addison disease. Hence, target tissue resistance or hypersensitivity to these hormones is also expected to be associated respectively with glucocorticoid deficiency or excess manifestations.

## Disclosure of interest

None declared.

